# Epitopia: a web-server for predicting B-cell epitopes

**DOI:** 10.1186/1471-2105-10-287

**Published:** 2009-09-14

**Authors:** Nimrod D Rubinstein, Itay Mayrose, Eric Martz, Tal Pupko

**Affiliations:** 1 Department of Cell Research and Immunology, George S Wise Faculty of Life Sciences, Tel Aviv University, Tel Aviv 69978, Israel; 2 Department of Zoology, University of British Columbia, Vancouver, BC, V6T 1Z4, Canada; 3 Department of Microbiology, University of Massachusetts, Amherst, MA 01003, USA

## Abstract

**Background:**

Detecting candidate B-cell epitopes in a protein is a basic and fundamental step in many immunological applications. Due to the impracticality of experimental approaches to systematically scan the entire protein, a computational tool that predicts the most probable epitope regions is desirable.

**Results:**

The Epitopia server is a web-based tool that aims to predict immunogenic regions in either a protein three-dimensional structure or a linear sequence. Epitopia implements a machine-learning algorithm that was trained to discern antigenic features within a given protein. The Epitopia algorithm has been compared to other available epitope prediction tools and was found to have higher predictive power. A special emphasis was put on the development of a user-friendly graphical interface for displaying the results.

**Conclusion:**

Epitopia is a user-friendly web-server that predicts immunogenic regions for both a protein structure and a protein sequence. Its accuracy and functionality make it a highly useful tool. Epitopia is available at  and includes extensive explanations and example predictions.

## Background

The detection of highly immunogenic regions within a given protein, specifically those that elicit a humoral immune response i.e., B-cell epitopes, is central to many immunodetection and immunotherapeutic applications [1,2]. An unguided experimental search for such regions is clearly laborious and resource-intensive. Thus, computational approaches that are able to perform this task are desired.

Extensive studies regarding the physico-chemical and structural aspects of antibody-antigen molecular recognition have provided several important characteristics of a typical epitope [3-6]. With this rich information at hand and the availability of state-of-the-art pattern recognition and classification algorithms, a computational tool that predicts the most antigenic regions in a protein, which can thus be approximated as immunogenic, is called for. Indeed, several such tools have been developed over the years. Some only rely on properties that can be extracted from the linear sequence of the antigen (ABCpred [7] and COBEpro [8]), while others rely on an available three-dimensional (3D) structure (CEP [9] and DiscoTope [10]). Other structure-based tools can be applied to linear sequences if a structural homolog can be found (ElliPro [11]). Yet, to date, no tool has been reported to perform its predictions either on the structure or directly on the sequence, if a structure is unavailable.

Here we present the Epitopia server, which implements a machine-learning based algorithm to predict immunogenic regions as candidate B-cell epitopes using either the 3D structure or the sequence of a given protein. We compare the performance of Epitopia to several other tools that either predict B-cell epitopes given a protein 3D structure or sequence alone and show that it has greater predictive power.

The Epitopia algorithm infers the immunogenic potential at the single amino-acid site resolution. Epitopia computes an immunogenicity score for each solvent accessible residue if a 3D structure was provided as input or a score for every amino-acid if a sequence input was provided. In addition, Epitopia combines a powerful visualization tool that color-codes the immunogenicity scores on either the protein sequence or the 3D structure to provide the users with a perceptible image of the immunogenic nature of their studied protein.

Herein we provide a short description of the Epitopia methodology. More detailed descriptions are available under the 'OVERVIEW', 'GALLERY', and 'QUICK HELP' web sections. We exemplify the use of Epitopia by predicting immunogenic regions for both a 3D structure and a sequence input. Finally, we report its performance on a benchmark dataset and compare it to other available tools.

## Implementation

The Epitopia algorithm [12] uses a Naïve Bayes classifier to predict the immunogenic potential of protein regions. The classifier was trained to recognize immunogenic properties using a benchmark dataset of 66 non-redundant validated epitopes derived from antibody-antigen co-crystal structures (an updated dataset compared to [11]), and 194 non-redundant validated epitopes derived from antigen sequences (for further reading about the data and immunogenic properties please refer to [13] and the 'OVERVIEW' web section, respectively).

A given antigen input is divided to overlapping surface patches (or stretches in the case of a linear sequence input), with the size of a typical epitope. Epitopia then computes for each patch (or stretch) the probability that it was drawn from the population of epitopes on which the classifier has been trained, with respect to each one of its physico-chemical and structural-geometrical properties. The immunogenicity score is thus the sum of logs of these probabilities and is assigned to the central residue of the patch (or to the middle residue in the linear stretch) [12].

The immunogenicity score reflects the immunogenic potential of a certain residue relative to all residues in the antigen. In order to have a more intuitive measure of immunogenic potential, we also provide a probabilistic score. To this end, we first divided all site-specific immunogenicity scores in the training data to quantiles (octiles for the structure data and noniles for the sequence data). For each quantile, we computed the fraction of validated epitope residues out of the total number of residues in the quantile. This number approximates the probability that a residue with a given immunogenicity score that falls in this quantile is an epitope residue.

We note that in structure-based predictions our method refers only to solvent exposed residues since, similar to other types of protein-protein interfaces, buried residues are not actively participating in the interaction. In cases where a studied protein may undergo cleavage which results with peptides that may become B-cell epitopes themselves [14], the 3D structure may not be relevant for the prediction and the sequence-based prediction should thus be used.

### Epitopia input

For a protein 3D structure input, Epitopia requires a protein data bank (PDB [15]) file (or its identifier), which can either be an X-ray crystal model or a representative NMR model of the protein of interest. In addition, the user should specify the relevant chains to which Epitopia should relate in one of the following options: (1) if all of the chains in the model should be related to, either all chain identifiers or the term "all" should be specified; (2) if only a subset of chains in the model should be related to, the corresponding chain identifiers should be specified. All non-selected chains will thus be removed from the model file in the preprocess stage; (3) the non-selected chains can be kept by marking the relevant checkbox. In this case, the structural-geometrical considerations for computing the immunogenicity scores will be affected by all the chains in the model, but immunogenicity scores will only be computed for the residues of the selected chains.

For a protein sequence input, the amino-acid sequence may either be pasted or a local sequence file can be uploaded. In either case, the sequence should be in Fasta format and should contain only standard amino acids.

The input is then preprocessed and several stand-alone executables are used to extract some of the physico-chemical and structural-geometrical properties required for Epitopia. Further details regarding the preprocess stage are available under the 'OVERVIEW' web section.

### Epitopia output

The immunogenicity and corresponding probability scores are computed by Epitopia for each surface residue for a 3D structure input or for every amino-acid for a sequence input. In either case, these scores are given as a text file link. In addition, the immunogenicity scores are color-coded and projected onto the protein. The visualization tool that is used for the 3D structure case is the FirstGlance in Jmol interface [16], which enables a wide range of display options. Along with that, Epitopia also provides a RasMol command script for viewing the results locally with the RasMol program [17].

For the sequence output case, an automatic search procedure for clustering highly immunogenic amino acids on the linear sequence is performed since it is not naturally evident as in the case of 3D structure output. Briefly, the clustering procedure divides the sequence to stretches and assigns each stretch a corresponding *p*-value, which is defined as the probability of randomly obtaining an equally-sized stretch with such a score or higher. The score of a stretch is the sum of immunogenicity scores of the amino acids comprising it. Practically, the *p*-value is computed by shuffling all the scores in the sequence and repeating the search procedure a large number of times. Eventually, these clusters, ranked according to their statistical significance (detailed in the 'OVERVIEW' web section) are given as a text file link.

## Results and discussion

### Case studies

**Figure 1:**
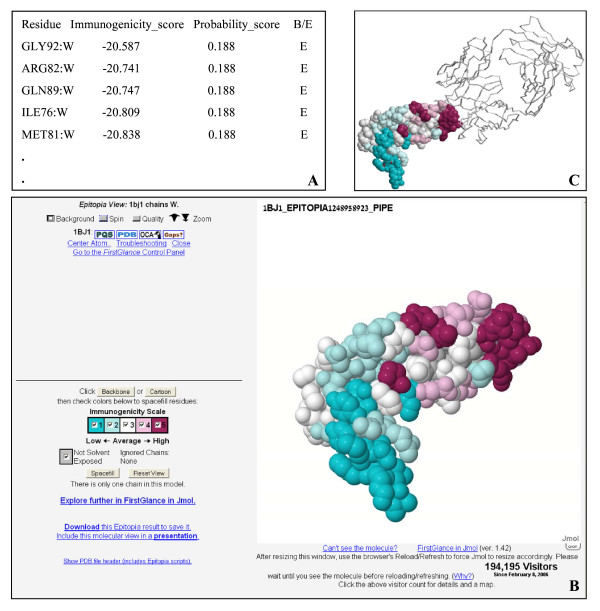
**Illustration of Epitopia's prediction for the 3D structure of the VEGF [PDB**: 1BJ1**, chain W]**. (A) A sample of the immunogenicity and probability scores computed for each of the surface residues of the input structure. (B) The FirstGlance in Jmol interface presenting the color-coded immunogenicity scores projected onto the surface of the protein shown in spacefill. (C) Presentation of the VEGF structure, color-coded as in A, along with the backbones of the chains of its binding antibody [PDB: 1BJ1, chains L and H].

To illustrate the performance and functionality of the Epitopia server two examples are given, one for a 3D structure input, and one for a sequence input. The 3D structure model is of the human vascular endothelial growth factor (VEGF), which was co-crystallized with its binding antibody (PDB: 1BJ1[18]). Figure 1 illustrates Epitopia's prediction, when only the VEGF chain of the complex (chain identifier W) was selected. The immunogenicity and probability scores (partly displayed in Figure 1A) are color-coded and projected onto the structure model using the FirstGlance in Jmol interface (Figure 1B). Figure 1C shows that the region predicted to be the most immunogenic largely overlaps the genuine epitope of the neutralizing antibody, making it a highly successful prediction. The FirstGlance interface further enables a wide range of display options for the graphical output such as increasing the display quality, zoom control, and different chain display modes.

**Figure 2:**
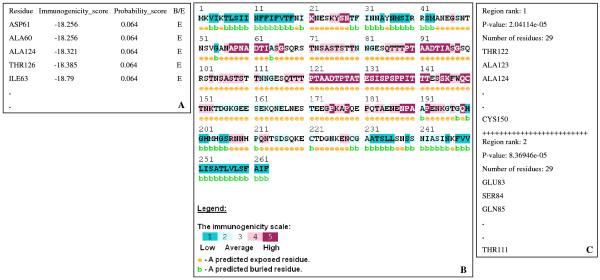
**Illustration of Epitopia's prediction for the sequence of the *P. falciparum *MSA-2 [Swiss-Prot**: P19599**]**. (A) A sample of the immunogenicity and probability scores computed for each amino acid of the input sequence. (B) Projection of the color-coded immunogenicity scores onto the protein amino-acid sequence. (C) A sample of the most significant immunogenic stretches obtained by the clustering procedure.

Figure 2 illustrates the prediction of Epitopia given the amino-acid sequence of the *Plasmodium falciparum *Merozoite surface antigen 2 (MSA-2) [Swiss-Prot: P19599]. Figure 2A presents a sample of the immunogenicity and probability scores computed for this sequence, where Figure 2B displays the graphic visualization of these scores color-coded and projected onto the sequence, along with the predicted surface accessibility status for each amino acid (whether it is buried or exposed). It is evident that the region spanning amino acids 121 to 142 is highly immunogenic. Correspondingly, the most significant immunogenic stretch according to Figure 2C lies between amino acids 122 and 150. According to the Bcipep database [19], a validated epitope for this sequence includes the stretch between amino acids 125 to 131.

### Comparing Epitopia to other B-cell epitope prediction tools

Conventionally, the area under the receiver operating characteristic (ROC) curve (AUC) [20] is used for diagnosing the performance of prediction methods (e.g., Ponomarenko and Bourne [21] used the AUC measure for evaluating several B-cell epitope prediction methods). Yet when it comes to assessing the performance of epitope prediction methods, the AUC is somewhat inadequate. In order to be able to compute the AUC, one has to define which residues are true epitope residues and which are non-epitope residues. It follows that any predictions which are not part of any validated epitope are regarded as false predictions. However, it is quite possible that the tested antigen harbors a far larger number of epitopes than are currently known, and thus the AUC underestimates the actual predictive power of the prediction method (this limitation was also noted by Ponomarenko et al., [11]). We thus consider an additional measure to evaluate the accuracy of prediction. Intuitively, in a successful prediction, genuine epitope residues should be scored higher than the average score of all residues. Hence, we considered a prediction (for a single protein input) to be successful if the average score of genuine epitope residues exceeds the average score of all considered residues. Accordingly, we define the success rate of a method as the number of successful predictions divided by the total number of predictions. Our method's parameters were optimized to achieve such maximal ratio. We also provide the AUC scores, which as noted above, provide a lower bound to the method's performance.

We compared Epitopia's performance to three other structure-based epitope prediction tools, CEP [9], DiscoTope [10], and ElliPro [11], on the same data and using exactly the same assessment measures. Epitopia succeeded in 59 out of the 66 predictions, yielding a success rate of 89.4%. In comparison, DiscoTope and ElliPro succeeded in 54 and 53 predictions, giving success rates of 81.8% and 80.3%, respectively. Since CEP does not individually score amino acids its performance could only be assessed using the AUC (computed as described in [21]). CEP achieved a mean AUC of 0.53 (over 65 cases, since a prediction for one of the datasets, PDB ID: 3FFD could not be obtained), which is substantially lower than that of all other methods (mean AUCs of 0.6, 0.62, and 0.59 for Epitopia, DiscoTope, and ElliPro, respectively).

Epitopia was additionally compared to two sequence-based tools, ABCpred [7] and COBEpro [8], which also implement machine-learning algorithms and were trained on very similar data as Epitopia. Epitopia succeeded in 156 out of 194 predictions (success rate = 80.4%) with a mean AUC of 0.59. ABCpred succeeded in 130 out of 194 predictions (success rate = 67%) with a mean AUC of 0.55. COBEpro succeeded in 119 out of 178 predictions (success rate = 66.9%), (16 antigen sequences were discarded since they exceed COBEpro's sequence length limit) with a mean AUC of 0.55.

We have selected the leave-one-out cross-validation procedure so that the performance of Epitopia is evaluated on data different from that used to train the classifier (thus avoiding over-fitting). In contrast, the performances of the methods to which Epitopia was compared were not achieved using cross-validation (thus, in most cases the compared classifier was trained and evaluated on the same data). Clearly, training and evaluating a method on the same data can artificially bias (increase) its performance.

## Conclusion

The Epitopia algorithm treats the problem of epitope prediction as a classical classification problem, applying the most suitable methodology for tackling it. To this end, Epitopia relies on an extensive set of physico-chemical and structural-geometrical features that characterize epitopes [6], which was optimized to yield maximal predictive power [12]. Although the Naïve Bayes classifier is often claimed to be over-simplified [22], we note that a support vector machine (SVM) classifier was also applied to this problem but did not perform as well as the Naïve Bayes classifier (data not shown). Thus, as the SVM classifier is claimed to be second-to-best for most of the classification problems, we feel that the Naïve Bayes classifier is an appropriate choice. Finally, it is worth emphasizing that the performance assessment measure defined here serves as a good alternative to the commonly used AUC measure, so long as the validated data remain scant. Although this new measure reports higher values than the AUC, it does so for all the compared methods without favoring any method in particular.

The Epitopia server provides ease of use, bifunctionality (in handling both 3D structure and sequence inputs), and rich output and visualization options that enable users to delve into the prediction results. These features along with the superiority of the Epitopia algorithm make up the main advantages of the Epitopia server over other related servers.

## Availability and requirements

Project name: Epitopia

Project home page: 

Operating system(s): Platform independent

Programming languages: C++, Perl

Any restrictions to use by non-academics: for non-commercial research purposes only

## Authors' contributions

NDR, IM, and TP conceived the algorithm. NDR developed the server. EM developed the graphical tool implemented in the server. NDR drafted the manuscript. All authors read and approved the final manuscript.
